# Selection of Competitive and Efficient Rhizobia Strains for White Clover

**DOI:** 10.3389/fmicb.2019.00768

**Published:** 2019-04-23

**Authors:** Pilar Irisarri, Gerónimo Cardozo, Carolina Tartaglia, Rafael Reyno, Pamela Gutiérrez, Fernando A. Lattanzi, Mónica Rebuffo, Jorge Monza

**Affiliations:** ^1^Laboratorio de Microbiología, Departamento de Biología Vegetal, Facultad de Agronomía, Universidad de la República, Montevideo, Uruguay; ^2^Instituto Nacional de Investigación Agropecuaria, INIA Treinta y Tres, Treinta y Tres, Uruguay; ^3^Laboratorio de Bioquímica, Departamento de Biología Vegetal, Facultad de Agronomía, Universidad de la República, Montevideo, Uruguay; ^4^Instituto Nacional de Investigación Agropecuaria, INIA Tacuarembó, Tacuarembó, Uruguay; ^5^Instituto Nacional de Investigación Agropecuaria, INIA La Estanzuela, Colonia, Uruguay

**Keywords:** white clover, *R. leguminosarum* sv. trifolii, nodule occupancy, inoculant, native-naturalized rhizobia strains, biological nitrogen fixation

## Abstract

The practice of inoculating forage legumes with rhizobia strains is widespread. It is assumed that the inoculated strain determines the performance of the symbiosis and nitrogen fixation rates. However, native-naturalized strains can be competitive, and actual nodule occupancy is often scarcely investigated. In consequence, failures in establishment, and low productivity attributed to poor performance of the inoculant may merely reflect the absence of the inoculated strain in the nodules. This study lays out a strategy followed for selecting a *Rhizobium leguminosarum* sv. trifolii strain for white clover (*Trifolium repens*) with competitive nodule occupancy. First, the competitiveness of native-naturalized rhizobia strains selected for their efficiency to fix N_2_ in clover and tagged with *gus*A was evaluated in controlled conditions with different soils. Second, three of these experimental strains with superior nodule occupancy plus the currently recommended commercial inoculant, an introduced strain, were tested in the field in 2 years and at two sites. Plant establishment, herbage productivity, fixation of atmospheric N_2_ (^15^N natural abundance), and nodule occupancy (ERIC-PCR genomic fingerprinting) were measured. In both years and sites, nodule occupancy of the native-naturalized experimental strains was either higher or similar to that of the commercial inoculant in both primary and secondary roots. The difference was even greater in stolon roots nodules, where nodule occupancy of the native-naturalized experimental strains was at least five times greater. The amount of N fixed per unit plant mass was consistently higher with native-naturalized experimental strains, although the proportion of N derived from atmospheric fixation was similar for all strains. Plant establishment and herbage production, as well as clover contribution in oversown native grasslands, were either similar or higher in white clover inoculated with the native-naturalized experimental strains. These results support the use of our implemented strategy for developing a competitive inoculant from native-naturalized strains.

## Introduction

Most legumes establish symbiosis with rhizobia, bacteria capable of fixing atmospheric nitrogen in a process referred to as biological nitrogen fixation (BNF). BNF allows saving nitrogenous fertilizers, with economic, and environmental benefits (Altier et al., 2013). The demand for sustainable agricultural practices has thus reinforced the attention in BNF (Sulieman and Tran, 2015), particularly in symbioses involving economically important food, and forage crops (Lindström et al., 2010).

Competition between inoculants and native strains for nodule occupancy is a widely recognized problem (diCenzo et al., 2018). Nitrogen fixation in an inoculated pasture is assumed to be due to the strain used in the inoculant, although which strains actually occupy the nodules is generally unknown. Red clover (*Trifolium pratense* L.) and white clover (*Trifolium repens* L.) are inoculated in Uruguay since 1967 with *Rhizobium leguminosarum* sv. trifolii strain U204 introduced from the United States (Labandera et al., 1982). In clovers, this practice is done because the soils have native-naturalized *R. leguminosarum* sv. trifolii strains without appropriate symbiotic efficiency. These less efficient indigenous rhizobia strains can be more competitive than the inoculant and occupy a significant portion of the nodules, reducing the impact of the inoculant strain on herbage production (Rodríguez et al., 2010; Yates et al., 2011). The commercial inoculant for white clover, as those used in Uruguay for lotus (*Lotus corniculatus* L.) and alfalfa (*Medicago sativa* L.), were selected decades ago (Lindström et al., 2010), for soil and management conditions different from the current ones. The displacement of cultivated pastures to marginal lands caused a decrease in the herbage production of clover. In Uruguay white clover is a widely used forage legume, both in ley farming pastures and oversown into native grasslands (Wilman et al., 2005), contributing substantially to the nitrogen budget of these systems (Mallarino and Wedin, 1990).

Batista et al. (2015), assaying red clover inoculation in different soils of Uruguay, confirmed that the U204 strain can be uncompetitive in soils with populations of native-naturalized rhizobia. Competitive strains are important for white clover because its primary root is relatively short lived, particularly in subtropical environments, being replaced by nodal roots emerging from stolons (Caradus, 1990). Thus, white clover persistence and efficient nitrogen fixation depends on rhizobia strains being able to survive in the soil and infect stolon roots. Further, in subtropical areas with climatic conditions marginal for this species, such as Uruguay (Real et al., 2005), white clover stands are typically lost during dry, and hot summers (García et al., 2010). Re-establishment from the seed bank is possible (Wilman et al., 2005), but again this requires competitive and efficient rhizobia strains able to survive in the soil to infect the new seedlings.

The competitiveness of a strain has become recognized as a key characteristic to consider when developing rhizobia inoculants (Schumpp and Deakin, 2010), particularly for soils with competitive but less efficient strains that are better adapted to local conditions (Estrella et al., 2009). Moreover, the evaluation of nodule occupancy necessary to know the competitiveness of a strain must include field assessments that confirm results from controlled conditions (Fabiano and Arias, 1991; Nangul et al., 2013). The advance of molecular tools has simplified this task, which currently can be done by the generation of genomic profiles (Svenning et al., 2001; Denton et al., 2002; Yates et al., 2005; Sotelo et al., 2011).

The aim of this study was to provide a proof-of-concept of a strategy for the development of suitable strains to be used as clover inoculants in Uruguay based on (i) the identification of efficient and competitive native-naturalized strains, followed by (ii) lab- and field-testing of their performance. For this, we evaluated nodule occupancy of native-naturalized strains of rhizobia selected for their efficiency in different soils, and then assessed strain effects on white clover establishment, nitrogen fixation, and herbage productivity.

## Materials and Methods

### Bacterial Strains and Forage Legume Seeds

In Uruguay, *R. leguminosarum* sv. trifolii U204 strain (Nitragin, United States, synonyms CIAT 2445, U-28) is the recommended and only commercially available inoculant for white clover and red clover. Strains N2, N5, 249, and 317, previously isolated in our laboratory from red clover nodules growing in soils without history of clover inoculation (Gutiérrez, 2017), were confirmed as symbionts of white clover. Rhizobia were long-term stored at -80°C in glycerol 25%, and grown in yeast extract-mannitol (YEM; Vincent, 1970).

*Escherichia coli* strain S17-1 λ-pir containing the plasmid pCAM131 that carries transposon mTn5SSgusA31 (Wilson et al., 1995) was grown in LB medium (Miller, 1972) at 37°C supplemented with spectinomycin and streptomycin.

Seeds of *T. repens* “Estanzuela Zapicán,” *T. pretense* “Estanzuela116,” *T. alexandrinum* “INIA Calipso,” *T. resupinatum* “Sirius,” and *T. vesiculosum* “Sagit” were stored at 4°C. Prior to use, seeds were surface disinfected with 90% (v/v) ethanol, followed by 4 min in 20% (v/v) NaClO, and rinsed three times in sterile distilled water. Seeds were germinated on sterile water-agar Petri dishes in the dark at 25°C. Plants were grown in a growth chamber at 22/20°C (day/night), with a 16/8 h photoperiod and 220 μE m^-2^ s^-1^ photosynthetic photon flux density.

### Estimation of Most Probable Number (MPN) of Rhizobia in Soil

Numbers of rhizobia were estimated by the MPN technique (Vincent, 1970), that has been validated by metagenomic, and metatranscriptomic datasets in soil (Mauchline et al., 2014).

White clover seedlings were planted in agar slants (15 mL per tube of N-free Jensen nutrient solution) in 25 mm × 250 mm glass tubes. After 3 days, plants were inoculated with serial tenfold dilutions of soil samples taken from the top 10 cm. The diluent contained NaCl 0.85% with 0.01% Tween 80 (Fisher Scientific Co.) added as a surfactant. Four tubes were used for each dilution. Plants were scored for nodulation 4 weeks after inoculation. Tables of Fisher and Yates (1963) were used to estimate the MPN of rhizobia.

### Assessment of Strains Competitiveness in Controlled Conditions

#### Tagging With gusA

The rhizobia strains were tagged with reporter gene *gus*A as described in Batista et al. (2015). Transconjugants were selected in YEM medium with antibiotics spectinomycin, streptomycin and nitrofurantoin to inhibit the growth of *E. coli* (150, 200, and 20 mg mL^-1^, respectively). Nodulation kinetics of the transconjugants were evaluated and compared with their parental strain. For this, seedlings of white clover inoculated with the parent strains or with the transconjugants were grown in tubes of N-free Jensen medium as described in Batista et al. (2015). Nodulation kinetics was evaluated since the appearance of the first nodule and for four more weeks. Nodulation rate was estimated as the slope of the linear regression of the number of nodules respect to time.

For detecting Gus activity, nodules were washed with distilled water for 15 min and placed in a Falcon tube with a solution type containing 1% SDS and 1 mM 5-bromo-4-cloro-3indolil-β-D-glucuronide (X-glucA) in 50 mM sodium phosphate buffer pH 7.5 (Wilson et al., 1995). The tubes were capped and held for 16 h at 37°C in darkness. Nodules occupied by the tagged bacteria were visually identified by internal blue staining, in contrast to those infected by non-tagged strains which remained unstained. The presence of mixed nodules was minimized recording only completely stained nodules as we did not detect partial staining (Sessitsch et al., 1997).

#### Assessment of Strains Tagged With gusA Competitiveness in Different Soils

Strains U204, N2, N5, 249, and their derivate *gus*A clones, were grown in YEM medium until they reached an OD600 of 0.9, corresponding to approximately 10^9^ cells mL^-1^. Then, these bacteria cultures were injected in bags containing a stabilization support based on sterile peat and incubated for 7 days at 25°C.

Seed inoculation was made at the recommended commercial doses (200 g of inoculum per 25 kg of white clover seed). Inoculated seeds were sown in pots (height 20 cm × diameter 8 cm) containing undisturbed soils from 5 sites of different agroecological regions of Uruguay (Table 1). The sites of the soils collection were different to those where the native-naturalized rhizobia strains were isolated. For each soil, two locations with and without history of clover inoculation were selected. Five pots (replicates) were used per strain and soil type. Plants were grown for 65 days and then, roots were washed with water, separated, and prepared for staining as described above. All the nodules present in each pot were analyzed.

**Table 1:** Soils used for the competitiveness test.

			Organic carbon	Total nitrogen
Site	Location	Type of soil	(%)	(%)	pH
Palo a Pique	33°15′S, 54°28′W	Typic Argiudoll	1.9	0.30	5.9
Glencoe	32°01′S, 57°09′W	Typic Hapludert	3.9	0.45	5.7
Cerro Colorado	33°51′S, 55°35′W	Typic Argiudoll	5.5	0.33	5.7
La Magnolia	31°41′S, 55°48′W	Typic Hapludarf	2.0	0.14	5.2
Cuchilla del Ombú	31°49′S, 55°36′W	Typic Argiudoll	5.6	0.26	5.7


*The sites of soil collection were selected because they have white clover pastures and for each soil there were locations with and without history of clover inoculation.*

### Assessment of Establishment and Herbage Production in Field Trials

To evaluate the performance of experimental strains in the field, trials were set up at INIA Experimental Unit “Palo a Pique” (33°15’S, 54°28’W) in the Lomadas del Este agroecological region, and at INIA Experimental Unit “Glencoe” (32°01’S, 57°09’W) in the Basalto agroecological region. These experimental sites were chosen because in the regions where the research stations are located there have been reported problems in the establishment and maintenance of white clover pastures. At the Palo a Pique site, the soil was a Typic Argiudoll with pH 5.9 and 1.9% organic C and 0.30% organic N contents in the top soil, with no history of clover inoculation. At Glencoe, the soil was a Typic Hapludert with pH 5.7 and 3.9% organic C and 0.45% organic N contents in the top, with strain U204 history of inoculation.

At the Palo a Pique site, white clover was oversown into a native grassland, whereas at the Glencoe site, it was sown in rows as a monoculture in a no-till bare bed. The experiments were repeated 2 years, 2015 and 2016; sowing dates were May 12, 2015 and June 10, 2016 at Palo a Pique, and April 15, 2015 and May 08, 2016 at Glencoe. At both sites, white clover was sown at rate of 4 kg seed ha^-1^ inoculated with the selected strains 317, N2, and 249. Further, a non-inoculated and inoculated with strain U204 treatments were added as controls. The inoculants were prepared in support peat as detailed in Batista et al. (2015). Plots of 3 m × 2 m at Experimental Unit Palo a Pique, and of 4 m × 2 m at Experimental Unit Glencoe were arranged in randomized complete block experimental designs with four replicates, and placed 0.6 m apart from each other to prevent contamination. The experiments were fertilized with 80 kg P_2_O_5_ ha^-1^.

#### Nodule Occupation and Biomass Allocation Between Organs

**FIGURE 1:**
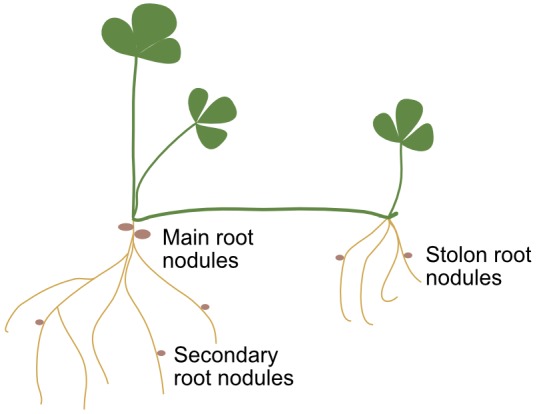
Diagram of white clover indicating the three types of roots where nodules occupation was evaluated: main or primary root, secondary roots, and nodal or stolon roots.

Ten soil cores 8 cm diameter and 10 cm depth with white clover plants were randomly harvested from each plot 7 months after sowing in each experiment for both sites. Roots were washed off soil, and nodules from all plants were counted and collected systematically, discriminating three root zones: the primary root, the secondary roots, and roots formed from nodes of stolons (Figure 1). At least 40 nodules per treatment and root type were stored at -20°C in glycerol until surface disinfection and rhizobia isolation in YEM medium (Vincent, 1970). Roots without nodules, leaves and stolon biomass were expressed as dry mater.

#### ERIC-PCR Genomic Fingerprinting

Isolated genomic DNA of the rhizobia strains was PCR amplified using the enterobacterial repetitive intergenic consensus (ERIC) primers ERIC1R and ERIC2 (de Bruijn, 1992) as described by Agius et al. (1997). The four strains of rhizobia studied presented different ERIC-PCR profiles (Supplementary Figure S1).

#### White Clover Establishment and Herbage Production

White clover establishment was assessed in both sites by counting the number of seedlings per square meter 60 days after sowing, in three areas of 0.1 m × 1 m per plot.

All biomass above 5 cm was harvested mechanically in a 2.5 m^2^ area whenever white clover height reached 10–15 cm. Herbage production was determined over two consecutive years, for two sowing years, at the Glencoe site; on average, plots were harvested 5–6 times per year. On the contrary, white clover did not reach the cutting height more than a few times in either of the two sowing years at the Palo a Pique site (data not shown). Harvested fresh matter was weighted, and dry matter content estimated in a subsample dried at 60°C in a forced-air oven for 72 h.

#### Nitrogen Fixation Measurement Using ^15^N Natural Abundance Method

The contribution of soil N uptake vs. fixation of atmospheric N to plant N acquisition was estimated 7 months after seeding with the ^15^N natural abundance technique (Oberson et al., 2007). This is based on the fact that the N isotopic composition [δ^15^N (‰) = (^15^N/^14^N_sample_)/(^15^N/^14^N_standard_) – 1) × 1000] of atmospheric N differs from that of N derived from soil organic matter (Högberg, 1997).

N concentration (% of dry matter) and isotopic composition were determined on samples of aerial part of plants (Huss-Danell et al., 2007). Leaves material was oven-dried, weighed, and ground to a fine powder (Sample Tek Model 200 Vial Rotator, Mavco Industries Inc., Kentucky, United States). Samples were placed in tin capsules, and analyzed in an elemental analyser (Flash EA 1112 series, Thermo Finnigan, Bremen, Germany) coupled to an isotopic ratio mass spectrometer (Delta Plus Thermo Finnigan, Bremen, Germany) via a ConFlo III interface.

The percentage of N derived from fixation of atmospheric N (%N_fix_) was estimated as:

(1) $$\%N_{\mathrm{fix}}=(\delta^{15}N_{\mathrm{plantref}}-\delta^{15}N_{\mathrm{plantfix}})/(\delta^{15}N_{\mathrm{plantref}}-B)\times 100$$

Where δ^15^N_plantfix_ is the δ^15^N of the legume sample, B is the δ^15^N of a plant whose N supply depends completely on atmospheric fixation, and δ^15^N_plant_ _ref_ is the δ^15^N of a non-nodulated plant whose supply depends completely on soil N. The value of B was estimated as -1.45 growing white clover in pots with sterile sand-vermiculite and watered with nitrogen-free Hoagland medium. The value of δ^15^N_plantref_ was measured on ryegrass plants (Hansen and Vinther, 2001) grown on the same soil and year (range from 2.0 to 6.0). Organic soil N at the Palo a Pique site had a δ^15^N of 7.4%, at Glencoe, a δ^15^N of 6.9%.

### Strains Efficiency in Other Clovers Used as Forage in Uruguay

Strains N2, 249, 317, and U204 were grown in YEM medium until they reached an OD_600_ of 0.9, corresponding to approximately 10^9^ cells mL^-1^. Seedlings of *T. pratense, T. alexandrinum, T. resupinatum*, and *T. vesiculosum* grown in pots with sand-vermiculite were inoculated as Batista et al. (2015). Five pots (replicates) were used per strain and legume.

### Statistical Analysis

The competitiveness of tagged rhizobia inoculants was assessed using a full factorial design with three factors (sites, inoculation history and rhizobia strains as fixed effects) with a completely random distribution and five replicates assumed as random effect. The significance of the effects of each factor was evaluated using a generalized linear mix model (GLMM) with binomial distribution for the variable number of blue nodules over the total number of nodules recorded and a logit link function.

In field trials, nodule occupancy was assessed separately for each site, year and root type with a random block distribution and four replicates, assuming random replicate effect, and strain fixed effects. The significance was evaluated using a GLMM with binomial distribution for the variable number of nodules occupied by the inoculants over the total number of nodules recorded and a logit link function. Strain effects on the number of plants established per m^2^ was assessed separately for each site and year of sowing, with each sampled area nested within the replicate, using a GLMM with correction of variance heteroskedasticity. The effects of the experimental inoculants over that of U204 on the accumulated biomass per year of sowing were analyzed using GLMM, where strain, days after sowing and their interactions were used as fixed effects and block, and plot nested to block as random effects. All data were analyzed using the statistical software InfoStat (Córdoba et al., 2014) and the multiple means comparison was done with the DGC test (exclusive group formation test; Di Rienzo et al., 2002).

## Results

### Competitiveness of Tagged Inoculants in Different Soils

The native-naturalized strains N5, N2, and 249, selected for their similar or higher nitrogen fixation efficiency than the commercial inoculant strain U204 (Supplementary Figure S2), were marked with *gus*A reporter gene. Since the insertion of the transposon containing the *gus*A gene occurs randomly in the genome of the recipient, three *gus*A clones derived from each strain were compared with their parental strains in relation to their nodulation kinetics in white clover. There was no difference in the time to first nodule appearance (7–9 days) or in nodulation rate (0.19–0.21 nodules per day).

A marked clon for each strain was inoculated in soils from five different agroecological regions (Table 1), each with and without history of clover inoculation with strain U204. Their MPN of rhizobia nodulating white clover were generally low, lesser than 100 g^-1^ of soil in all but soil from the Palo a Pique site with inoculation history, that was 350 g^-1^ of soil (Supplementary Table S1).

Nodule occupancy by the inoculants tagged with *gus*A varied with soil origin (Table 2) and no interaction between soil site and strain was detected. For all native-naturalized strains, nodule occupancy was always higher (*p* < 0.05) in soils without previous history of clover inoculation than in soils with history (Table 3). In contrast, nodule occupancy was lower for commercial inoculant strain U204 than for the native-naturalized strains (Table 3), independently of the inoculation history.

**Table 2:** Nodules occupancy by rhizobia tagged with *gus*A under controlled conditions in undisturbed soils from sites belonging to different agroecological regions of Uruguay.

Soil type	Nodules occupancy (%)
La Magnolia	70 a^∗^
Palo a Pique	40 b
Cuchilla del Ombú	40 b
Cerro Colorado	26 c
Glencoe	23 c


*^∗^Different letters indicate significant differences (DGC test *p* < 0.05).*

**Table 3:** Nodule occupancy (%) by rhizobia strains tagged with *gus*A under controlled conditions in undisturbed soils with and without history of inoculation with the commercial strain U204.

	Inoculation history^∗^		
		
Inoculant	Without	With	Overall average^∗∗^
N2::*gus*A	76 a	47 b	62 a
249::*gus*A	71 a	48 b	60 a
N5::*gus*A	77 a	37 b	57 a
U204::*gus*A	18 c	8 c	13 b


*Values correspond to the mean of % of nodules occupied in five soil types. ^∗^Different letters indicate significant differences for the interaction of inoculation history and inoculant strain (DGC test *p* < 0.05). ^∗∗^Different letters indicate significant differences within the column (DGC test *p* < 0.05).*

The nodule occupancy of each marked rhizobium strain in each agroecological zone is reported in Supplementary Table S2.

### Competitiveness of the Inoculants in the Field

The two sites selected for the field experiments belong to the agroecological zones where a decrease of forage production had been detected after the first year of clover sowing (Rodríguez et al., 2010). They also represent two of the main uses of white clover in Uruguay, at Palo a Pique to improve natural grasslands and at Glencoe as a no-till culture.

The number of clover rhizobia previous to sowing was similar in both sites (50 and 30 g^-1^ of soil at Palo a Pique and Glencoe, respectively). Nodule occupancy for the experimental strains was higher or similar to that of U204 in both years and sites evaluated, and for all root types, except for strain 249 in nodules of the primary root at Palo a Pique in 2015 (Table 4).

**Table 4:** Nodule occupancy (%) by different inoculant strains in 7 months old white clover plants, sown at two sites (Glencoe and Palo a Pique), and in two years (2015 and 2016), in primary root (PR), secondary roots (SR), roots formed from nodes of stolons (StR), and whole plant average (A).

	Palo a Pique				Glencoe			
		
	PR	SR	StR	A	PR	SR	StR	A
**Inoculant**	**Sowing 2015**							
	
317	87 a**^∗^**	58 a	47 a	63 a	60 a	42 a	73 a	58 a
N2	91 a	73 a	68 a	77 a	80 a	51 a	51 a	69 a
249	39 b	71 a	79 a	63 a	78 a	68 a	77 a	74 a
U204	61 a	17 b	9 b	30 b	58 a	53 a	013 b	42 a

	**Sowing 2016**							
	
317	100 a	100 a	77 a	91 a	56 b	57 b	76 a	64 a
N2	100 a	100 a	84 a	93 a	59 b	80 a	76 a	69 a
249	83 a	67 b	38 b	53 b	69 a	87 a	53 b	62 a
U204	58 b	55 b	5 c	36 c	50 b	32 b	57 b	49 b


***^∗^**Different letters indicate significant differences within each site, year, and location of nodules [PR, SR, StR, and average (DGC *p* < 0.05)].*

Differences were larger in secondary roots than primary roots and even larger in nodal roots than in secondary roots. Nodule occupancy by strain U204 was generally lesser than the other strains when the average of all types of roots nodules were considered, although the difference was not always significant (Table 4).

### Plant Establishment, Biomass Allocation, and N Fixation

Clover establishment was in general improved by inoculation in both sites and sowing years (Table 5). However, strain effects were not consistent and their magnitude variable. Inoculation with experimental strains 317 and 249 achieved greater densities of established plants than U204 at three out of four experiments, with an average effect of 31%. Strain U204 only surpassed native-naturalized experimental strains in one site and one year (Palo a Pique sowing 2015).

**Table 5:** Establishment of inoculated white clover, measured as number of plants per m^2^, at two sites (Palo a Pique and Glencoe) in two consecutive years (2015 and 2016).

	Palo a Pique		Glencoe	
		
Inoculant	2015	2016	2015	2016
317	118 b^∗^	85 a	107 b	301 c
N2	115 b	80 a	123 b	234 d
249	67 c	77 a	166 a	406 a
U204	141 a	44 b	82 c	213 d
Control	76 c	30 c	70 c	346 b


*A non-inoculated control was included. ^∗^Different letters indicate significant differences for each site and year after MLGMix (Poisson)-DGC with *p* < 0.05.*

Biomass allocation between organs in 7-months old plants was similar for white clover inoculated with different strains, except for stolons (Table 6). Inoculation with strain 317 resulted in heavier stolons at both sites, whereas inoculation with N2 and 249 strains were only superior in one site (Palo a Pique and Glencoe, respectively). Plants were generally larger at the Glencoe site.

**Table 6:** Biomass allocation between organs in 7 months old white clover plants at Palo a Pique and Glencoe sites.

	Palo a Pique			Glencoe		
		
Inoculant	Root	Leaf	Stolon	Root	Leaf	Stolon
N2	0.94 a^∗^	0.49 a	1.20 a	3.22 a^∗^	1.56 a	5.35 b
317	1.15 a	0.53 a	1.28 a	4.27 a	2.38 a	7.05 a
249	0.76 a	0.31 a	0.87 b	4.33 a	2.01 a	7.26 a
U204	0.68 a	0.36 a	0.68 b	3.73 a	2.43 a	6.29 b
Control	0.31 b	0.15 b	0.81 b	3.55 a	2.24 a	6.13 b


*^∗^Different letters indicate significant differences for each component and site after MLMix (DGC with *p* < 0.1).*

The proportion of nitrogen derived from N_2_ fixation in 7-months old plants was similar between strains at both sites (Table 7). Average values were higher at the Palo a Pique site than at the Glencoe site (97 vs. 81%, respectively). The N concentration and the amount of N fixed per unit plant mass (calculated considering N concentration) were consistently higher with native-naturalized experimental strains than with U204 strain (Table 7).

**Table 7:** Nitrogen concentration, percentage of N derived from N_2_-fixation, and N fixed per unit of plant dry matter of 7-months-old white clover plants inoculated with different rhizobia strains at two sites (Palo a Pique and Glencoe).

	N concentration (%)			N fixed (%)			Kg Nfix/Mg DM		
			
Strain	Palo a Pique	Glencoe	Mean^∗^	Palo a Pique	Glencoe	Mean^∗^	Palo a Pique	Glencoe	Mean^∗^
N2	3.5	3.0	3.3 a	96	84	90	34	25	30 a
249	3.3	3.1	3.2 a	99	87	93	33	27	30 a
317	3.4	2.9	3.1 a	94	79	86	32	23	27 a
U204	2.9	2.6	2.8 b	98	73	85	29	19	24 b
Mean^∗^	3.3 a	2.9 b		97 a	81 b		32 a	23 b	


*^∗^Different letters indicate significant differences for either each strain or site after MLMix (DGC with *p* < 0.05).*

### Herbage Production

At the Palo a Pique site, where white clover was oversown into native grassland, total herbage production was very low due to harsh climatic conditions. Thus, in sowing year 2015, only 8 months of herbage production could be assessed, and in sowing year 2016 there was no assessment on herbage production over the first year. For this reason, annual herbage production is not presented nor analyzed for this site.

**FIGURE 2:**
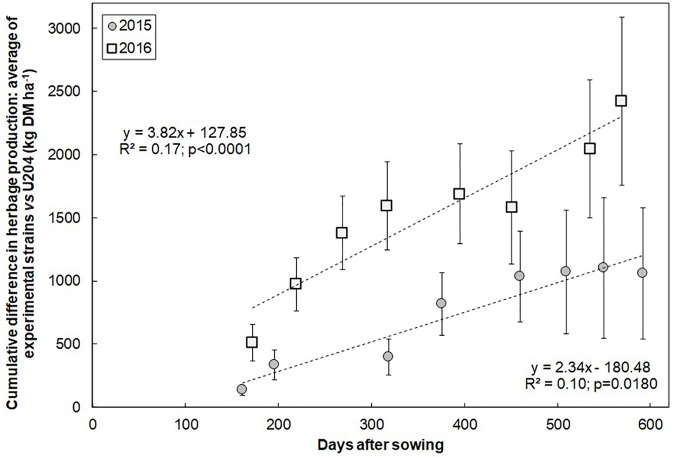
Cumulative difference in herbage production of the native-naturalized rhizobia strains (N2, 249, and 317) vs. commercial inoculant strain (U204) along two years for two consecutive sowings (2015 and 2016). Doted gray lines represent the regression for each year.

Annual white clover herbage production at Glencoe was moderate for sowing year 2015 and high for sowing year 2016: 4400 and 7100 kg DMha^-1^year^-1^, respectively. White clover comprised 40–90% of the produced herbage for sowing year 2015, and 60–100% in sowing year 2016, the rest being weeds. In both sowing years, for most harvest dates, white clover herbage production was higher when inoculated with native-naturalized strains than with strain U204. Nonetheless, we were not able to detect most of these differences as statistically significant (Supplementary Table S3). However, this response resulted in a continuously increasing difference in accumulated herbage production between native-naturalized strains and U204, which was detected as statistically significant (Figure 2).

### Efficiency of the Experimental Strain in Other Clovers

The symbiotic efficiency of the rhizobia strains in red clover, *T. resupinatum, T. alexandrinum*, and *T. vesiculosum* was estimated as dry matter of aerial part 30 days after seeding. The strain was considered Fix^-^when the dry aerial biomass was equal to the control without inoculation and Fix^+^ if it was heavier. The experimental strains and U204 were Fix^-^ in *T. vesiculosum* and Fix^+^ in red clover, *T. resupinatum* and *T. alexandrinum* (Supplementary Table S4).

## Discussion

In the rhizobia-legume symbiosis, plants can form root nodules with multiple rhizobial strains present in the soil (Denison and Kiers, 2004). In crops inoculated with commercial strains, it is often assumed that the inoculated strain determines the performance of the symbiosis and nitrogen fixation rates. However, in the presence of competitive native-naturalized strains actual nodule occupancy by the inoculant strain can be low (e.g., in other clovers: Denton et al., 2002; Nangul et al., 2013; Batista et al., 2015). This has important practical implications for agriculture, for differences in nitrogen fixation efficiency between strains can be large and are, indeed, the basis for the selection of elite inoculants (Dwivedi et al., 2015). Therefore, understanding the interaction between native and commercial rhizobia strains, in relation to the host plant preference, is relevant for the development of effective and competitive strains (Checcucci et al., 2017).

Competitiveness for nodule occupancy and efficiency of nitrogen fixation are distinct traits (Bourion et al., 2018). Hence, there is a need for a selection strategy that includes strain competitiveness to insure that rhizobial strains suitable as inoculants are also able to colonize the soil, tolerate environmental stresses, and compete with background rhizobia populations to form nodules (Slattery and Pearce, 2001). In this study we provide proof of concept of one such strategy for white clover, based on the selection of native-naturalized rhizobia strains and involving assessments under both controlled conditions and in the field.

### Competitiveness of White Clover Native-Naturalized Rhizobia Strains

Nodule occupancy of the experimental strains and the commercial inoculant was assessed using soils from five regions where white clover is sown and have contrasting agroecological characteristics (Table 1). The MPN of rhizobia of these soils (Supplementary Table S1) were generally low (10–100 rhizobia g^-1^ soil), according to the criterion established by Martyniuk and Oroń (2008).

The ratio of inoculant‘s nodule occupancy was more than twice in soils without history of clover cultivation than in soils with history (Table 3). This assessment served to demonstrate, first, that soil previous inoculation history affected nodule occupancy (Yates et al., 2011). Further, native-naturalized strains were more competitive than U204, the commercial inoculant, across all soil types and inoculation history (Tables 2, 3). Batista et al. (2015), working on red clover, had already reported similar results.

Soil type may influence the outcome of competition between strains because the rhizospheric and saprophytic competitiveness depend on the degree of adaptation of rhizobia to local soil conditions reviewed by Onishchuk et al. (2017). Busby et al. (2017) affirmed that plant genotype × environment × microbiome × management interactions are the challenges to the success of beneficial microbes in agricultural management. However, we found no interaction between soil type and strain in nodule occupancy under these conditions and in the short term, which may be positive for the strategy proposed, as it shows that there is no need to recommend specific strains for specific soils.

The responses observed under controlled conditions were confirmed when competitiveness was assessed under field conditions, which provide more realistic conditions and allow longer term evaluation. A lower occupation of nodules by the commercial inoculant respect to soil population rhizobia has already been reported in clover species (Denton et al., 2002; Duodu et al., 2007; Nangul et al., 2013). In the present study, the advantage of the native-naturalized strains in nodule occupancy compared to strain U204 was most evident in nodal roots, that is, in roots produced from stolons (Table 4).

The establishment of efficient nodal roots nodules is essential for white clover persistence, since the primary tap root of the plant rarely lives more than one year under subtropical conditions (Caradus, 1990). Nodule occupancy in these roots depend entirely from strain ability to persist in the soil and outcompete local rhizobia populations. On the other hand, in indeterminate nodules like those of clover, bacteroids are terminally differentiated and only bacteria not differentiated may be released to the soil from decaying nodules at the end of symbiosis (Wielbo et al., 2010). These rhizobia liberated of senescent primary root nodules should contribute to nodal roots occupation. There were 5.6 times more nodal roots nodules occupied by the experimental strains than by U204 (Table 4). These results further confirm inferences made in controlled conditions, demonstrating that these native strains are well adapted to different conditions, have a suitable fit with the soil and rizosphere microbiomes and more chances to nodulate stolon nodal roots. To our knowledge, such detailed results had not been reported previously.

The existence of native-naturalized strains with nitrogen fixation efficiency similar to that of elite commercial strains is a prerequisite for the strategy proposed by the present study to be successful. Therefore, a central question is how likely is that such strains exist? This may depend primarily on the history of inoculation, as transfer to native strains of symbiotic genes located in plasmids of elite *R. leguminosarum* strains may be one mechanism. In field conditions, natural transfer of symbiotic islands has in fact been shown in mesorhizobia, where resident non-nodulating bacteria accepted symbiotic genes from the inoculant strain and formed new nodulating species (Nandasena et al., 2007; Sotelo et al., 2011). Some of the native strains of rhizobia used in the present study share the same symbiotic genes with U204 but in different chromosomal backgrounds (Tartaglia et al., 2019).

### Effects of Rhizobia Strains on Plant Performance

The next step of the strategy was to corroborate whether native strains were not only more competitive than the commercial one, but also able to enhance plant performance in the field. The population of rhizobia in different soils are heterogeneous and vary quantitative and qualitatively responding to different abiotic and biotic factors (Graham, 2008). Our field trials represented two contrasting situations where clover is usually sown in Uruguay. Both soils had similar pH, but their organic C content may have allowed the proliferation of different rhizobial populations capable of catabolizing different available substrates (Rynne et al., 1994). Further, at Palo a Pique site, clover was oversown into a native grassland and thus had to compete with an established stand of grasses.

Plant establishment was improved by inoculation (Table 5), but this depended on the site and year of sowing considered. Factors other than a high initial density of rhizobia determined seedling establishment success in these trials.

Conversely, the amount of N fixed per unit of above ground plant biomass was consistently higher for native-naturalized strains across sites than the commercial strain U204 (Table 7), indicating a greater capacity of these strains to provide N to the host, and compared to the U204 inoculation. N fixed per unit plant biomass combines the effects of strain on the proportion of N derived from biological fixation and on N concentration on plant biomass. It should be noted that even though the proportions of biological fixation was equal across strains, the total amount of biological fixation of the native strains was higher than the total amount fixed by the commercial strain (e.g., 90% BFN of 30 Kg Nfix/Mg DM is still larger than 85% BFN of 24 Kg Nfix/Mg DM). The higher soil derived N content could be the result of the higher plant vitality and root production caused by the beneficial rhizobia.

White clover inoculated with the different rhizobia strains showed similar proportions of N derived from biological fixation (94–99% at Palo a Pique, and 73–87% at Glencoe; Table 7), values similar to those observed in mixed white clover/grass pastures in Uruguay (Mallarino and Wedin, 1990). The difference between sites would result from differences in soil type (organic C and N, Table 1), or more likely, in sowing conditions. At Palo a Pique, oversown white clover plants had to compete for soil N with well adapted native C4 grass species. At Glencoe, white clover was the dominant or only species, and thus could capture soil N. Negative relationships between clover proportion in pastures and proportion of N derived from biological fixation are common (Mallarino et al., 1990), and the fact that legumes prefer to use soil N or fertilizer when available instead of more costlier biological N fixation is well documented (Ledgard and Giller, 1995; Burchill et al., 2014).

The efficiency to fix N of a rhizobium strain is determined by its genetic and physiology in interaction with the legume host and the edaphological and climatic conditions (Weaver and Wright, 1987). Plants inoculated with the native strains had higher N concentration (Table 7) in the aboveground parts, and also higher herbage production (Figure 2). The strain N2 showed the most consistent response along the two years of each of the two sowing dates. These responses could derive from the greater mass of stolons (Table 6) and the higher nodule occupancy of stolon root-nodules (Table 4) observed in white clover plants inoculated with the native-naturalized strains.

Native strains showed the same behavior as the commercial inoculant with other clover species (Supplementary Table S4). This characteristic is a prerequisite for the selection of elite inoculant as rhizobia fitness varies dependent of the host species (Pahua et al., 2018).

### Strategies for Selection of Rhizobia Strains for Inoculant Development

**FIGURE 3:**
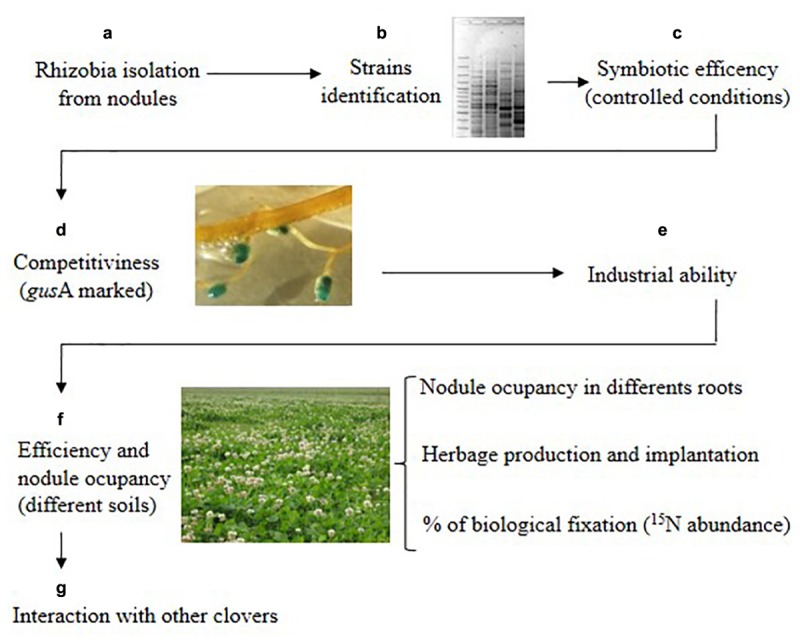
Strategy to select competitive and efficient native-naturalized rhizobia strains for white clover used in this work. Letters represent the different stages of the strategy.

Microbial-based strategies that improve forage legume productivity have long been exploited through rhizobial inoculation technology. The importance of a continued effort to identify and select rhizobia strains with higher nitrogen-fixing capacity has been underlined (Lindström et al., 2010). Masson-Boivin and Sachs (2018) reviewed the molecular mechanisms underlying the rhizobium-legume symbiosis and divided them in those implicated in nodule formation and invasion and those involved in nitrogen fixation. Ideal candidates’ strains to be used as elite inoculants in agriculture must have both characteristics, so to prevent outcompetition of symbiotically effective strains by indigenous rhizobial population that can be less efficient in nitrogen fixation. In this study, we provided a proof of concept for a strategy (outlined in Figure 3) to carry out such selection.

It comprises the following stages: (a) Collection of rhizobia obtained from red nodules of old pastures; (b) Identifying the rhizobial isolates (ERIC-PCR for example); (c) Screening the symbiotic efficiency in controlled condition; (d) Assess the competitiveness for nodule occupation of the strains in different soils in controlled conditions (marked strains with *gus*A for example); (e) Check the industrial ability of the strain; (f) Field evaluation of the selected strains at different sites and years, including pasture establishment, herbage production, N_2_-fixation and nodules rhizobia occupation; (g) Test the compatibility of the strains with other clover hosts.

## Conclusion

This study provides proof of concept for a strategy to select inoculants with high competitive ability under field conditions for regions with introduced exotic legumes and their symbionts. Native-naturalized rhizobia strains selected by their N fixation efficiency, showed greater nodule occupancy than the currently used commercial introduced strain, independent of soil type, and of previous history of inoculation. Most importantly, the differences in nodule occupancy between the selected native-naturalized strains and the commercial one remained when assessed under field conditions at two sites and in two sowing years, being greatest for nodules from stolons. These organs, that are essential for white clover performance as they replace primary roots and thus white clover persistence *via* vegetative propagation, depend entirely on soil rhizobia for its nodulation. Plant establishment, herbage production and N fixed per unit plant biomass were either similar or higher when white clover was inoculated with the experimental native-naturalized strains than with the currently used commercial strain.

## Author Contributions

JM and MR conceived the project. GC and RR executed the field assays and PG and CT laboratory experiments. PI wrote the first draft of the manuscript. FL rewrote it. All authors contributed to the statistical analysis, manuscript revision, and final version.

## Conflict of Interest Statement

The authors declare that the research was conducted in the absence of any commercial or financial relationships that could be construed as a potential conflict of interest.
