# Supplementary material for: Selection of Competitive and Efficient Rhizobia Strains for White Clover
Source: Front Microbiol. 2019 Apr 23;10:768. doi: 10.3389/fmicb.2019.00768 (PMC6489563; doi:10.3389/fmicb.2019.00768)
Supplement: Supplementary file 1 [file Table_1.DOCX]

Supplementary Material

**Selection of competitive and efficient rhizobia strains for white clover**

**Irisarri P., Cardozo G.A., Tartaglia C., Reyno R., Gutiérrez P., Lattanzi F.A., Rebuffo M., Monza J.^*^**

*** Correspondence:** Corresponding Author: jmonza@fagro.edu.uy

## Supplementary Figures


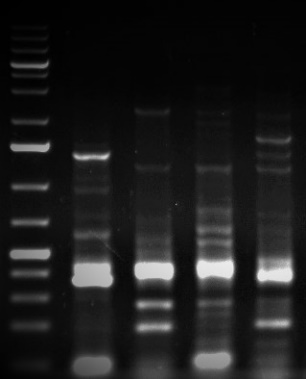


A B C D E

**Supplementary Figure 1.**ERIC-PCR electrophoresis profiles of the *R. leguminosarum* sv. *trifolii* used in this study. A. Molecular weight marker; B. U204; C. 249; D. N2; E. 317.

**Supplementary Figure 2.** Symbiotic efficiency in white clover of native-naturalized rhizobia strains isolated from red clover nodules (Batista et al., 2015) and the commercial inoculant U204 (dark grey). Treatment 0 (black) corresponded to a non-inoculated treatment, and selected strains are shown in white. Symbiotic efficiency is expressed as dry matter of the aerial part of 60 days-white clover plants grown in sand-vermiculite.

## SupplementaryTables

**Supplementary Table S1.**Rhizobia most probable number (MPN) per gram of soil in different sites with and without U204 inoculation history.

|  | History of inoculation | |
| --- | --- | --- |
| Site | Without | With |
| Palo a Pique | 16 c* | 350 a |
| Glencoe | 43 b | 70 b |
| Cerro Colorado | 6 c | 9 c |
| La Magnolia | <1 c | 1 c |
| Cuchilla del Ombú | 63 b | 90 b |

*Different letters indicate significant differences (DGC test *p*<0.05).

**Supplementary Table S2.** Nodules occupancy (%) by rhizobia tagged with *gus*A under controlled conditions in undisturbed soils from sites without and with history of inoculation with the commercial strain U204 belonging to different agroecological regions of Uruguay.

| Soil type | Inoculation history | N2::*gus*A | 249::*gus*A | N5::*gus*A | U204::*gus*A |
| --- | --- | --- | --- | --- | --- |
| Glencoe | Without | 23 b | 23 b | 9 b | 1 a * |
|  | With | 88 a | 51 a | 73 a | 7 a |
| La Magnolia | without | 82 a | 85 a | 90 a | 25 a |
|  | With | 85 a | 75 a | 77 a | 52 a |
| Palo a Pique | without | 69 a | 36 b | 31 b | 8 a |
|  | With | 71 a | 81 a | 77 a | 9 a |
| Cuchilla del Ombú | without | 54 a | 64 a | 34 b | 5 a |
|  | With | 71 a | 72 a | 79 a | 10 a |
| Cerro Colorado | without | 8 b | 33 b | 22 b | 0 a |
|  | With | 64 a | 74 a | 81 a | 12 a |

*Different letters indicate significant differences within the column (DGC test *p*<0.05).

**Supplementary Table S3.** Pure white clover biomass at Glencoe for sowings 2015 and 2016 for inoculated treatments and the non-inoculated control.

|  | Sowing 2015 (kg DM ha^-1^) | | | Sowing 2016 (kg DM ha^-1^) | | |
| --- | --- | --- | --- | --- | --- | --- |
| Inoculant | 1^st^ year | 2^nd^ year | 1^st^ year | | 2^nd^ year |  |
| N2 | 1298 a* | 5040 a | 6098 a | | 6624 a |  |
| 317 | 1422 a ǂ | 4989 a | 5743 a | | 6761 a |  |
| 249 | 1145 a | 4863 a | 6302 a | | 6718 a |  |
| U204 | 890 a | 4326 a | 4453 b | | 5871 a |  |
| Control | 847 a | 4302 a | 5758 a¥ | | 6276 a |  |

*Different letters indicate significant differences for each year and seeding after MLMix-DGC with p<0.05.

ǂ Different to U204 (p<0.1) by contrasts.

¥ Not different to U204 (p<0.1) by contrasts

|  | ***T. resupinatum*** | ***T. alexandrinum*** | ***T. vesiculosum*** |
| --- | --- | --- | --- |
|  | ----------------------**mg DM of a plant**------------------------ | | |
| **N2** | 0,09a | 0,18a | 0,01b |
| **249** | 0,10a | 0,17a | 0,02b |
| **317** | 0,09a | 0,09a | 0,01b |
| **U204** | 0,12a | 0,22a | 0,01b |
| **Control** | 0,03b | 0,03b | 0,02b |

**Supplementary Table S4.** Symbiotic efficiency estimated as plant dry matter (DM) of the different inoculants for three clover species (n=5).

*Different letters in each column indicate significant differences (DGC test *p*<0.05).
